# Are machine learning models superior to logistic regression models to predict 30-day mortality post-hip fracture surgery?

**DOI:** 10.1093/jbmrpl/ziag079

**Published:** 2026-04-28

**Authors:** Zeinab Issa, Fouad Trad, Ryan Yammine, Caline Rhayem, Aya Ghosn, Joudie Alwan, Hani Tamim, Rachid Haidar, Ali Chehab, Ghada El-Hajj Fuleihan

**Affiliations:** Division of Endocrinology, American University of Beirut Medical Center, Beirut 1107 2020, Lebanon; Electrical and Computer Engineering Department, American University of Beirut, Beirut 1107 2020, Lebanon; Electrical and Computer Engineering Department, Lebanese American University, Byblos P.O. Box 36, Lebanon; Division of Endocrinology, American University of Beirut Medical Center, Beirut 1107 2020, Lebanon; Calcium Metabolism and Osteoporosis Program, WHO Collaborating Center for Metabolic Bone Disorders, American University of Beirut Medical Center, Beirut 1107 2020, Lebanon; Division of Endocrinology, American University of Beirut Medical Center, Beirut 1107 2020, Lebanon; Division of Endocrinology, Memorial Sloan Kettering, New York, NY 10065, United States; Department of Pediatric Emergency Medicine, Children’s Hospital of Michigan, Detroit, MI 48201, United States; Department of Internal Medicine, Clinical Research Institute, American University of Beirut Medical Center, Beirut 1107 2020, Lebanon; College of Medicine, Alfaisal University, Riyadh 11533, Saudi Arabia; Division of Orthopedic Surgery, American University of Beirut Medical Center, Beirut 1107 2020, Lebanon; Electrical and Computer Engineering Department, American University of Beirut, Beirut 1107 2020, Lebanon; Division of Endocrinology, American University of Beirut Medical Center, Beirut 1107 2020, Lebanon; Calcium Metabolism and Osteoporosis Program, WHO Collaborating Center for Metabolic Bone Disorders, American University of Beirut Medical Center, Beirut 1107 2020, Lebanon

**Keywords:** hip fracture, mortality prediction, machine learning, logistic regression, risk stratifications, NSQIP

## Abstract

Hip fractures represent a significant global health burden, with high mortality rates. Accurate prediction of 30-d postoperative mortality is instrumental to optimize patient care. This study aimed to validate previously developed logistic regression and machine learning (ML) models for predicting 30-d mortality after hip fracture surgery measured from the date of the index operation (counting deaths occurring in-hospital or within 30 d after surgery) and compare their performance against one established calculator. We included 47 276 patients aged ≥65 yr who underwent hip fracture surgery, excluding cancer, atypical, pathologic, and stress fractures. We applied previously derived logistic regression and ML equations from the 2011 to 2017 National Surgical Quality Improvement Program (NSQIP) dataset, to the updated 2018-2020 dataset. We developed 2 models for each, one derived on admission and one taking into account operative course. For each of these models, we also developed a full model and a parsimonious counterpart. We assessed discrimination using the area under the curve (AUC) metric. We compared AUCs for models with the DeLong test. We developed an online calculator for our most highly performing model. We also benchmarked our preoperative logistic and ML calculators against a previously published preoperative model. The logistic models demonstrated acceptable to good discrimination with AUCs as follows: preoperative full of 0.756, preoperative parsimonious of 0.748, postoperative full of 0.829, and postoperative parsimonious of 0.817. Machine learning models maintained good discrimination performance: preoperative full, 0.772; preoperative parsimonious, 0.751; postoperative full, 0.865; and postoperative parsimonious, 0.838. Machine learning models were slightly superior to logistic regression models. A previously published model by Harris et al. had more variables and demonstrated no significant superiority. Both logistic regression and ML approaches offer clinically useful predictions for 30-d mortality after hip fracture surgery. Prospective validation in other populations and then integration of the parsimonious models into clinical practice may enhance perioperative decision-making and possibly patient outcomes.

## Introduction

Hip fractures remain a major global health concern, predominantly affecting the elderly population, with over 10 million cases occurring worldwide each year.^1^^,^^2^ Beyond their high prevalence, hip fractures impose a substantial socioeconomic burden on both patients and healthcare systems, including prolonged hospitalizations, extensive rehabilitation needs, and an increased risk of long-term disability and mortality.^3^ Within the first year, mortality rates can be as high as 20%-24%, with the initial month being the most critical.^4^ For survivors, the impact on independence is substantial: 40% lose the ability to walk independently, 60% still require assistance after a year, and one-third become fully dependent or enter nursing homes within the first year.^4^ Men face a particularly grim outlook, with first-year mortality rates reaching up to 37%, nearly double that of women.^4^ Given these challenges, accurate prediction of 30-d mortality is critical for guiding clinical decision-making and optimizing resource allocation, be it on admission or postoperative. In our previous study, we capitalized on the National Surgical Quality Improvement Program (NSQIP) dataset, a variable-based data registry from the American College of Surgeons designed to improve hospital-wide quality across all surgical departments, to develop risk score calculators using logistic regression models^5^ and machine learning (ML) models.^6^ External validation is essential to assess a prediction model’s reproducibility and generalizability, particularly as patient demographics and treatment protocols evolve through time.^7^ Without such validation, models remain theoretical tools rather than offering clinically applicable solutions.

Thus, the overall primary objective of this study is to compare the performance of various models,^5^^,^^6^^,^^8^ including our own (NSQIP 2011-2017 dataset), using a more recent dataset (NSQIP 2018-2020). For both logistic regression and ML approaches, we validated 2 model types: a preoperative model based on admission data and postoperative model that incorporated intra and postoperative information. Each of these models (pre-op and post-op) was evaluated in both a full form, including all significant variables, and more parsimonious versions, where only a few variables are considered.

Our aims in this study were to:

(1) Validate our hip fracture 30-d mortality logistic regression and ML models originally derived from the NSQIP 2011-2017 dataset in the more recent NSQIP 2018-2020 dataset.
(2) Compare the performance of our logistic regression models to the ML models, with all the permutations; preoperative full and parsimonious, postoperative full and parsimonious.
(3) Compare the validated preoperative logistic regression and ML calculators to the preoperative calculator developed by Harris et al.,^8^ using NSQIP 2011-2017 dataset.
(4) Deploy the model that best balances performance and parsimony as an online calculator for public use.

## Materials and Methods (flow diagram Figure S2)

### Eligibility criteria

Our eligibility criteria are similar to the ones of the original papers.

1. Inclusion criteria:

- Men and women aged 65 yr or above who underwent surgery for a hip fracture (either femoral neck fracture via open reduction and internal fixation (ORIF) or total hip or bipolar hip hemiarthroplasty or inter/peri/subtrochanteric femoral fracture via ORIF or intramedullary fixation), as per defined current procedural terminology (CPT) and International Classification of Diseases (ICD) codes (Appendix 1).

2. Exclusion criteria:

- Cancer patients.
- Patients with atypical femoral, pathologic, and stress fractures.

### Dataset selection and preparation

We obtained the NSQIP 2018-2020 orthopedic dataset as data from subsequent years (2021-2022) could not be included due to the removal of several key variables by NSQIP programmers that were required by our calculators, such as >10% weight loss, dyspnea, and emergency case status.^5^^,^^6^ We selected the same CPT and ICD codes of the original papers (Appendix 1). All preoperative and postoperative variables were obtained directly from the NSQIP dataset as available publicly, definitions, timing/collection windows are summarized in Table S1, we performed data cleaning and recoded some variables to ensure expression format was identical to the original dataset. Some continuous variables were categorized, such as BMI and preoperative hematocrit. We used standard descriptive statistics. Continuous variables are expressed as mean ± SD; categorical variables are expressed as counts and proportions (%). Statistical significance was defined a priori as a 2-sided alpha level of .05 (*p* < .05).

For all models, we used one common dataset that had complete data for all predictors used in the logistic regression and for the ML complete preoperative and postoperative models. Each patient/case appears only once in our analytic file; we verified uniqueness during the merge and retained a single record per case. We defined 30-d mortality as death from any cause within 30 d of the index operation. The time zero was the date/time of surgery; patients were followed for 30 d regardless of discharge status. In-hospital deaths and post-discharge deaths within 30 d were both counted as events; survivors were censored at day 30.

### Missing data

We initially explored ACS-NSQIP 2018-2022 and assessed missingness only for predictors used in our logistic and ML calculators and the Harris model. The variable weight loss (>10% in the prior 6 mo) showed the greatest missingness (38%), because it was no longer recorded in 2021-2022; therefore, we restricted the validation cohort to NSQIP 2018-2020 to ensure consistent availability of required predictors.

In the candidate validation cohort (*N* = 68 517), missingness was present for a limited set of predictors: BMI category (9029/68 517; 13.18%), preoperative International Normalized Ratio (INR) (9931/68 517; 14.49%), preoperative WBC (422/68 517; 0.62%), preoperative hematocrit (400/68 517; 0.58%), American Society of Anesthesiologists (Physical Status Classification) (ASA) classification (132/68 517; 0.19%), and anesthesia type (25/68 517; 0.04%). All other predictors had 0 missing. Records with missing predictor data were excluded using complete-case analysis, resulting in a final analytic validation cohort of 47 276 patients, as shown in the flow diagram in the Appendix. A detailed per-predictor missingness summary is provided in Table S3.

### Validation strategy

This is an external temporal validation of previously derived models: equations and ML fits from NSQIP 2011-2017 were applied without refitting to NSQIP 2018-2020. No *k*-fold or bootstrap cross-validation was performed on the 2018-2020 data.

### Logistic regression and machine models validation

We validated the logistic and ML models trained on the NSQIP dataset from 2011 to 2017^6^ on NSQIP data from 2018 to 2020. We assessed both the full-feature models and their parsimonious counterparts to evaluate their performance on unseen data and determine how they withstand temporal changes. In ML models, two features from the original models, DNR status and postoperative coma were unavailable in the 2018-2020 dataset. However, these 2 variables had a very low impact in the original project, with importance ranking 34th and 45th out of 45, respectively. For this reason, these variables were removed from our models. For the ML models, we selected the highest performing models as established in the original dataset.^6^ It was Adaboost for the preoperative model and Catboost for the postoperative model,^6^ both of which are ensemble ML methods that combine multiple decision trees to improve predictive performance.

### Comparing AUCs of all calculators

To compare the discriminative performance of the different preoperative and postoperative models, we used the DeLong test^9^ for correlated receiver operating characteristic (ROC) curves. The DeLong test is a nonparametric method that estimates the variance of the area under the curve (AUC) difference between 2 models, allowing for statistically rigorous comparison of their performances on the same dataset.^9^ We computed *p*-values to assess whether the observed differences in AUCs between the best-performing model and each of the remaining models were statistically significant. Separate comparisons were conducted for the preoperative and postoperative models. These comparisons were performed to identify the model with the most robust and significantly superior predictive performance in each clinical setting. Because the DeLong test compares two models at a time, we applied it pairwise across all model combinations. This pairwise approach allows identification of the best-performing model among multiple candidates by comparing it sequentially with each of the other models, and the resulting *p*-values are presented to characterize relative model performance. We did not apply formal multiplicity adjustment; results should be interpreted accordingly.

### Feature importance and variable ranking

For the logistic regression models, variable importance was determined based on the absolute magnitude of the standardized regression coefficients (β coefficients), with larger absolute values indicating greater influence on the predicted outcome. Variables were ranked accordingly to identify the most influential predictors. For the ML models, variable importance was derived from model-specific feature importance metrics reflecting each variable’s contribution to prediction performance. Since with ML only tree-based ensemble models are used, importance was quantified based on the relative contribution of each feature to reducing prediction error across the ensemble. Variables were ranked by their normalized importance scores to identify the highest-impact predictors. Separate rankings were generated for preoperative and postoperative models, as well as for full and parsimonious model variants.

### Case-based comparison of different calculators

To explore how the different calculators perform in practical use and how their risk predictions vary for individual patients, we applied each model to a sample of patient cases. Specifically, we purposefully selected 20 patients from the NSQIP dataset to represent a balanced and diverse range of clinical scenarios. The sample included 10 patients who died and 10 who survived, with equal representation of sex (5 men and 5 women in each outcome group). All selected patients were aged 70 yr or older. This subgroup was chosen to illustrate the calculators’ output across a spectrum of real-world cases and to assess how predicted risk scores may differ between models in clinically relevant situations. To facilitate interpretation, we stratified patients into 3 risk categories based on thresholds commonly used in post-cardiac surgery risk stratification: low risk (<4%), intermediate risk (≥4% to <8%), and high risk (≥8%).^10^

### Online calculator

We selected the best performing preoperative model for deployment (on-line tool) based both on its discriminative performance and parsimony. It does not include variables that were removed from NSQIP releases after 2020, such as >10% weight loss, dyspnea, and emergency case status. This online tool is intended for practical use with current NSQIP-compatible inputs, whereas the external validation in this manuscript was performed using the original model equations developed from the 2011 to 2017 cohort^5^ into the current 2018-2020 cohort, when all required predictors were still available. The deployed model prioritized predictive accuracy while minimizing input burden, requiring only a limited number of easily obtainable variables to facilitate clinical usability. This model was implemented as a web-based tool, designed for intuitive use by researchers and clinicians without the need for specialized software or extensive data entry. The calculator is made publicly accessible to support decision-making in real-world preoperative settings.

### Comparison to previously published calculator

We tested the Harris et al. preoperative calculator (2011-2017) in the 2018-2020 dataset using the beta estimates from their original Lasso model and derived an AUC.

### Calibration assessment (external validation)

In the external validation cohort, the calibration was evaluated for the logistic preoperative parsimonious model and the deployed ML model using calibration intercept and calibration slope with 95% CIs. Calibration was assessed by fitting a logistic regression model with the observed outcome as the dependent variable and the model’s linear predictor (logit of predicted probability) as the independent variable; the resulting intercept and slope quantify systematic under/over-prediction (ideal intercept = 0) and over-/under-fitting of predictions (ideal slope = 1). Calibration was visualized with calibration plots comparing observed event rates vs mean predicted probabilities across deciles of predicted risk.

## Results

### Baseline characteristics

We included a total of 47 276 hip fracture patients from the NSQIP 2018-2020 database in this validation study (Table 1). Their mean age was 82 (7) yr, 69% were females, 73% were White. Comorbidities were as follows: 10% smoked, 35% were overweight and 16% obese, 19% were diabetic, 31% hypertensive, and 11% had a history of severe chronic obstructive pulmonary disease (COPD); 78% were functionally independent. Differences in characteristics between males and females were statistically significant for all variables except functional health status and hypertension requiring medication. Women were slightly younger, more likely to be white, to have a lower BMI if outside the normal range, were less likely to smoke, to have diabetes, and had a slightly lower hematocrit, and operative time. The baseline characteristics of the 2011-2017 and 2018-2020 cohorts were for the most part comparable. However, we observed some notable shifts in surgical management over time. Compared to the 2011-2017 cohort, there was a decrease in ORIF procedures (from 12% to 6%) and partial hip arthroplasty (from 16% to 11%), while intramedullary fixation became more prevalent in the 2018-2020 cohort (from 38% to 47.2%). Despite these differences, the general patient profiles remained consistent across both time periods.

**Table 1:** Baseline characteristics of patients undergoing hip fracture surgery from the Orthopedic NSQIP 2018-2020 dataset.

**Variables (continuous)**		**Male *N* (%) 14 532 (31%)**	**Female *N* (%) 32 744 (69%)**	**Total *N* (%) 47 276 (100%)**	***p*-value (male vs female)**
		**Mean (SD)**			
**Age of patient (years)**		81 (8)	82 (7)	82 (7)	<.001
**Preoperative hematocrit (%)**		35.9 (6)	35.1 (5)	35.3 (5)	<.001
**Preoperative serum creatinine (mg/dL)**		1.3 (1.02)	1.0 (0.64)	1.1 (0.80)	<.001
**Total operation time (min)**		68.5 (40)	66.0 (37)	66.8 (38)	<.001
**Variables (categorical)**		**Male *N* (%) 14 532 (31%)**	**Female *N* (%) 32 744 (70%)**	**Total *N* (%) 47 276 (100%)**	***p*-value (male vs female)**
**Race**					
**Black or African American**		693 (5)	1095 (3)	1788 (4)	<.001
**Others**		423 (3)	1068 (3)	1491 (3)	.04
**Unknown**		2870 (20)	6349 (20)	9219 (20)	.36
**White**		10 546 (72)	24 232 (74)	34 778 (73)	.001
**Variables (categorical)**		**Male *N* (%) 14 532 (31%)**	**Female *N* (%) 32 744 (69%)**	**Total *N* (%) 47 276 (100%)**	***p*-value (male vs female)**
**BMI (kg/m** ^**2**^ **)**	**<18.5**	722 (5)	3110 (9.5%)	3832 (8)	<.001
	**18.5**-**24.9**	6413 (44)	16 094 (49)	22 507 (48)	<.001
	**25**-**29.9**	5086 (35)	8633 (26)	13 719 (29)	<.001
	**>=30**	2311 (16)	4907 (15)	7218 (15)	.011
**Smoker within 1 yr**	**No**	12 752 (88)	29 864 (91)	42 616 (90)	<.001
	**Yes**	1780 (12)	2880 (9)	4660 (10)	<.001
**Diabetes**	**No**	11 272 (78)	27 037 (83)	38 309 (81)	<.001
	**Yes**	3260 (22)	5707 (17)	8967 (19)	<.001
**Hypertension requiring medication**	**No**	4491 (31)	10 079 (31)	14 570 (31)	.79
	**Yes**	10 041 (69)	22 665 (69)	32 706 (69)	.79
**History of severe COPD**	**No**	12 669 (87)	29 473 (90)	42 142 (89)	<.001
	**Yes**	1863 (13)	3271 (10)	5134 (11)	<.001
**Congestive heart failure in 30 d before surgery**	**No**	13 727 (94)	31 649 (97)	45 376 (96)	<.001
	**Yes**	805 (5)	1095 (3)	1900 (4)	<.001
**Bleeding disorders**	**No**	11 301 (78)	27 808 (85)	39 109 (83)	<.001
	**Yes**	3231 (22)	4936 (15)	8167 (17)	<.001
**Functional health status prior to surgery**	**Independent**	11 315 (78)	25 433 (78)	36 748 (78)	.65
	**Partially dependent**	2767 (19)	6217 (19)	8984 (19)	.90
	**Totally dependent**	450 (3)	1094 (3)	1544 (3)	.17
**Anesthesia**	**General**	11 842 (81)	26 148 (80)	37 990 (80)	<.001
	**Neuraxial**	2690 (18)	6596 (20)	9286 (20)	<.001
**Type of surgery**	**IM fixation**	6467 (44)	15 829 (49)	22 296 (47)	<.001
	**Partial hip arthroplasty**	1752 (12)	3467 (11)	5219 (11)	<.001
	**ORIF femoral neck fracture**	4654 (32)	9787 (30)	14 441 (30)	<.001
	**ORIF inter/sub/peri**-**trochanteric femoral fracture**	856 (6)	1997 (6)	2853 (6)	.40
	**Total hip arthroplasty**	803 (5)	1664 (5)	2467 (5)	.045

Percentages and SDs in the table were rounded appropriately.

### Validation of preoperative mortality prediction logistic regression models

The full preoperative model incorporated 16 variables (same as parsimonious model in addition to ascites, bleeding disorder, type of surgery, steroid use, type of anesthesia, and diabetes as predictors). It achieved an AUC of 0.756 (0.743-0.768) (*R*^2^ = 0.9836) in the updated dataset. The parsimonious preoperative model, consisted of 10 key predictors (male gender, age, lower BMI, white race, poorer functional health status, higher creatinine, lower hematocrit, >10% weight loss in the past 6 mo, congestive heart failure within 30 d before surgery, and COPD). It achieved an AUC of 0.748 (0.736-0.760) (*R*^2^ = 0.9855) in the 2018-2020 cohort (Table 2). The AUCs of various models based on the 2011-2017 dataset are reported in Table S2. The calibration (*n* = 47 276) showed a calibration intercept of −0.336 (95% CI: −0.444 to −0.228) and calibration slope of 1.009 (95% CI: 0.963-1.055). The calibration plot by deciles demonstrated close agreement between observed and predicted risks, with mild overall over-prediction (negative intercept) Figure S3.

**Table 2:** Area under the curve (AUC) and CI for the different parsimonious models for using NSQIP 2011-2017 and NSQIP 2018-2020 datasets.

**Model (*N* variables)**	**NSQIP 2011**-**2017 *N* subjects 73 291**	**NSQIP 2018**-**2020 *N* subjects 47 276**
**Classic logistic preoperative parsimonious (10)**	0.74 (0.73, 0.75)	0.75 (0.74, 0.76)
**Classic logistic postoperative parsimonious (11)**	0.80 (0.79, 0.81)	0.82 (0.81, 0.83)
**ML preoperative parsimonious (10)**	0.77 (0.73, 0.82)	0.75 (0.74, 0.77)
**Deployed (on-line) preoperative ML model (15)**	0.78 (0.74, 0.82)	0.77 (0.76, 0.78)
**ML postoperative parsimonious (11)**	0.86 (0.83, 0.89)	0.84 (0.83, 0.85)
**Harris et al. (15)**	0.76 (0.75, 0.76)	0.75 (0.74, 0.76)

Harris et al., see reference.^8^

### Validation of postoperative mortality prediction logistic regression models

The full postoperative model incorporated 27 variables (same as parsimonious with the addition of renal failure, race, sepsis, >10% weight loss, preoperative hematocrit, ascites, readmission, pulmonary embolism, type of surgery, operation time, bleeding disorder requiring transfusion, steroid use, unplanned reoperation, type of anesthesia, and hypertension). It exhibited the highest predictive performance with an AUC of 0.829 (0.819-0.839) (R^2^ = 0.998). The parsimonious postoperative model included 11 predictors (all preoperative predictors except white race, hematocrit, and >10% weight loss, with the addition of unplanned intubation, cerebrovascular accident, myocardial infarction, and pneumonia). It achieved an AUC of 0.817 (0.806-0.828) (*R*^2^ = 0.999) (Table 2).

### Validation of preoperative mortality prediction ML models

**Figure 1:**
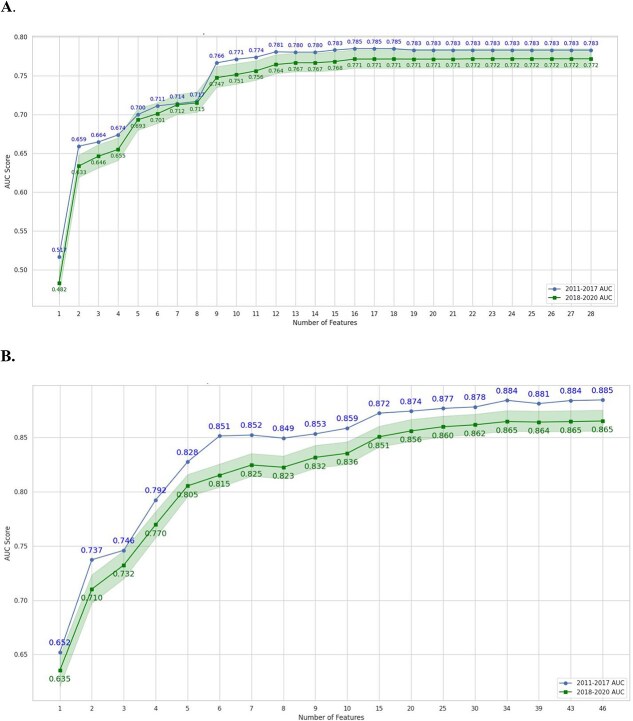
(A) Validation of the preoperative ML model on the new dataset with varying number of features. (B) Validation of the postoperative ML model on the new dataset with varying number of features.

The best-performing preoperative model (Adaboost) used 28 variables and maintained a high AUC of 0.772 (0.760-0.785). Similarly, the parsimonious models with fewer features also retained strong performance as shown in Figure 1A. For example, the 10-variable model, which achieved an AUC of 0.771 on the original data, recorded 0.751 (0.739-0.765) on the validation data (Table 2).

### Validation of postoperative mortality prediction ML models

The best-performing postoperative model (Catboost), which originally achieved an AUC of 0.885 with 45 variables, demonstrated continued excellence with an AUC of 0.865 (0.856-0.875) on the validation dataset. The parsimonious models also exhibited strong performance as shown in Figure 1B. For instance, the 15-variable model, which achieved an AUC of 0.872 on the original dataset, maintained an AUC of 0.850 (0.841-0.860) on the validation data (Table 2).

### Validation of the preoperative calculator by Harris et al. in the 2018-2020 dataset

We tested the Harris et al. preoperative calculator in the 2018-2020 dataset using the beta estimates from their original Lasso model (Code available in Appendix 1). As part of variable harmonization, we created recoded versions of NSQIP variables to match the specifications of each calculator; in some instances, the same underlying NSQIP variable was duplicated and recoded differently to meet calculator-specific definition. Their calculator used the 2011-2017 dataset and included 15 predictors: male gender, age, BMI, >10% weight loss, diabetes, functional health status, dyspnea, COPD, CHF, systemic sepsis, steroid use, bleeding disorder, hemodialysis, active cancer, and type of surgery (Table 2). This yielded an AUC of 0.749 (0.737, 0.761) compared to the 0.76 published (Table 3).

**Table 3:** Area under the curves (AUCs) and *N* of variables derived from the validation dataset NSQIP 2018-2020 for the various models.

	**Preoperative full AUC *N* of variables**	**Preoperative parsimonious AUC *N* of variables**	**Postoperative full AUC *N* of variables**	**Postoperative parsimonious/AUC *N* of variables**
**Classic logistic** ^**a**^	0.76 16	0.75 10	0.83 27	0.82 11
**Lasso Harris et al.** ^**b**^	N/A^a^	0.749/17	N/A	N/A
**ML**	0.77 28	0.75 10	0.87 46	0.84 11
***p* values** ^**c**^	ML vs Classic <.001	ML vs Classic .55 Harris vs classic .57 Harris vs ML .78	ML vs Classic <.001	ML vs Classic <.001

a *R* ^2^ values were available for the classic models and are reported here for completeness (not shown in the table to improve readability): preoperative full 0.983, preoperative parsimonious 0.985, postoperative full 0.998, and postoperative parsimonious 0.999.

b Harris et al., see reference.^8^

c *p*-values derived using the DeLong test (see Materials and methods).

### Comparison between different models

**Figure 2:**
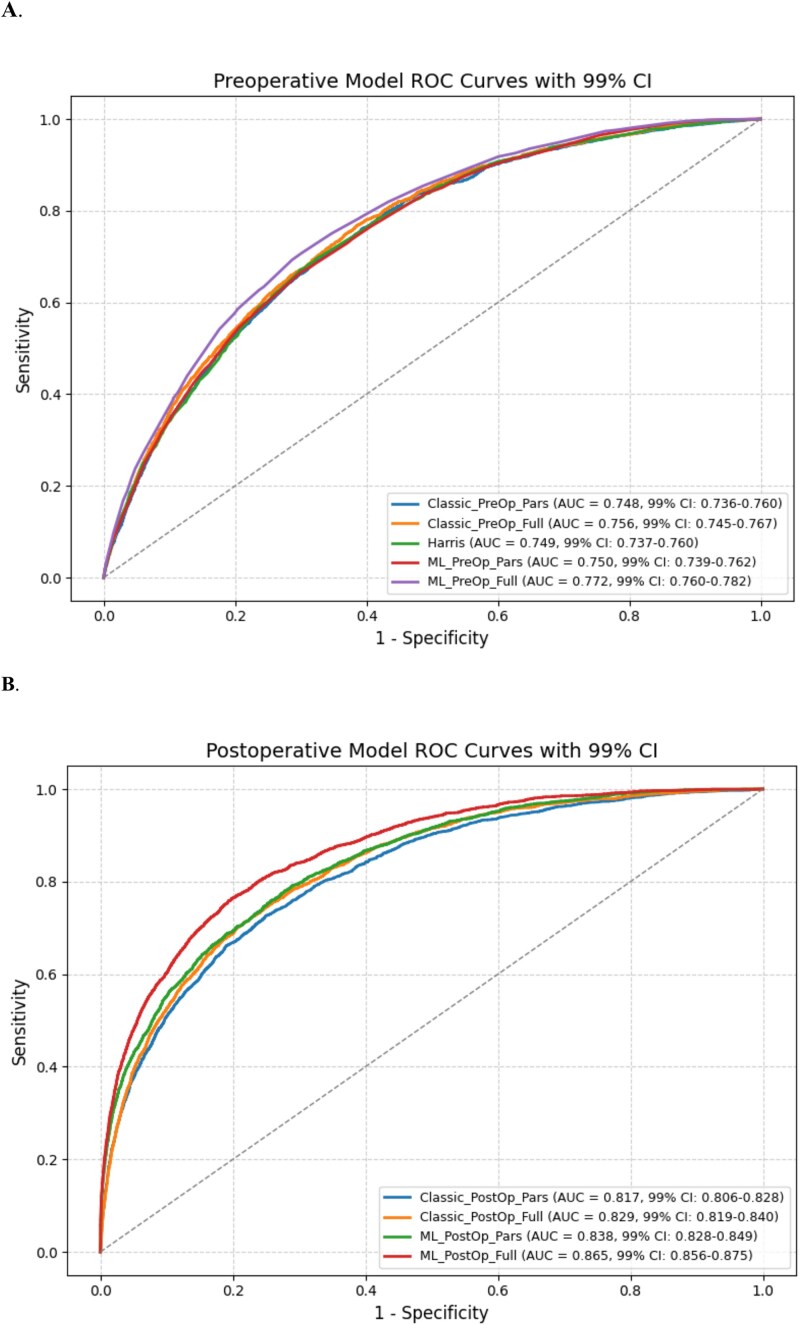
(A) Area under the ROC curve for the various preoperative models. (B) Area under the ROC curve for the various postoperative models.

Figure 2A displays the ROC curves for the preoperative models, including both full and parsimonious versions. Figure 2B presents the ROC curves for the postoperative models in both full and parsimonious forms. We compared the predictive performance (AUC) of the logistic regression models, the Harris model, and the ML models using the DeLong test. The corresponding *p*-values for all pairwise comparisons are shown in the heatmap in Figure S1. The ML models demonstrated significantly higher AUC values compared to classic logistic models, that is, logistic and Lasso, across all scenarios (*p* < .001). This was except for the preoperative parsimonious models, where performance was equivalent (*p* < .55) (Table 3). Interestingly, our 2 parsimonious models also performed just as well as the 17-variable Lasso model from Harris et al. (Harris vs classic P 0.57, Harris vs ML P 0.78) (Table 3).

### Comparison of major predictors by order of importance

Our analysis of eight models identified key variables associated with 30-d mortality (Table 4). In the classic logistic-regression calculators, the same clinical themes dominate across both the full and parsimonious versions. In the preoperative models (full and parsimonious), age and functional health status are the 2 strongest predictors, followed by gender, serum creatinine, COPD, BMI, and congestive heart failure, >10% weight loss and race. After surgery, the emphasis shifts: in both postoperative classic logistic models (full and parsimonious) the most influential variable is unplanned intubation, followed by age and functional status which remains prominent. Acute complications (pneumonia, stroke, and myocardial infarction) plus gender, creatinine and BMI comprise the remainder of the top-ranked factors.

**Table 4:** Major top predictors (up to 16 for full models as applicable) by order of importance for the NSQIP 2011-2017 dataset.

**Classic preoperative full (16)**	**Classic preoperative parsimonious (10)**	**ML preoperative full (28)**	**ML preoperative parsimonious (10)**	**Classic postoperative full (27)**	**Classic postoperative parsimonious (11)**	**ML postoperative full (46)**	**ML postoperative (11)**
**AUC 0.742**	**AUC 0.739**	**AUC 0.792**	**AUC 0.776**	**AUC 0.813**	**AUC 0.800**	**AUC 0.885**	**AUC 0.860**
**Age**	Age	Weight	Weight	Unplanned intubation	Unplanned intubation	Age	Age
**Functional health status**	Functional health status	Age	Age	Age	Age	ASA classification	ASA classification
**Gender**	Gender	Height	Height	Functional health status	Functional health status	Functional health status	Functional health status
**Pre-op creatinine**	Pre-op creatinine	Pre-op WBC	Pre-op WBC	Pneumonia	Pneumonia	Cardiac arrest requiring CPR	Cardiac arrest
**COPD**	COPD	Functional health status	Functional health status	CVA/stroke	CVA/stroke	Post-op pneumonia	Post-op pneumonia
**BMI**	BMI	Pre-op platelet	Pre-op platelet	MI	MI	Hospital length of stay	Hospital length of stay
**CHF**	CHF	Pre-op hematocrit	Pre-op hematocrit	Gender	Gender	Pre-op BUN	Pre-op BUN
**>10% weight loss**	>10% weight loss	Pre-op Na	Pre-op Na	Pre-op create	Pre-op creatinine	Weight	Weight
**Race**	Race	ASA classification	ASA classification	BMI	BMI	Reintubation	Reintubation
**Pre-op hematocrit**	Pre-op hematocrit	Type of surgery	Type of surgery	CHF	CHF	Pre-op INR	Pre-op INR
**Ascites**		Pre-op INR		COPD	COPD	Readmission	Readmission
**Bleeding disorder**		Pre-op BUN		Renal failure		Female sex	
**Type of surgery**		Female sex		Race		CDMI	
**Steroid**		Pre-op sepsis		Sepsis		Pre-op Na	
**Type of anesthesia**		COPD		>10% weight loss		CNS CVA	
**Diabetes**		CHF		Pre-op hematocrit		Height	

Abbreviations: ASA, American Society of Anesthesiologists Physical Status Classification; CDMI, chronic diabetes mellitus insulin-dependent; CNS, central nervous system; COPD, chronic obstructive pulmonary disease; CVA, cerebrovascular accident.

By contrast, the ML models weigh anthropometrics and granular labs more heavily. In the preoperative ML calculators (full and parsimonious), the leading predictors are weight, age, and height, followed by functional status and hematologic values (WBC, platelet count, and hematocrit) together with renal and electrolyte markers (creatinine and sodium) and ASA class. In the postoperative ML models (full and parsimonious) age remains paramount, but ASA class and functional status rise in importance, while early critical events like cardiac arrest requiring CPR and postoperative pneumonia plus length of stay, preoperative BUN, weight, BMI, and re-intubation make up the rest of the high-impact feature set.

The greater emphasis placed by ML models on anthropometric and laboratory variables likely reflects their ability to capture nonlinear relationships and complex interactions among continuous predictors. Variables, such as weight, height, and laboratory values, may encode subtle physiological risk gradients that are less readily represented in traditional regression frameworks, which rely on linear assumptions and prespecified variable effects. In contrast, logistic regression models may preferentially highlight clinically salient, categorical predictors that have strong and well-established associations with mortality.

### Sample of 20 patients

Table 5 provides a summary of the clinical features of the 20 patients along with their calculated mortality risk. The mortalities were coded into three colors low risk in light grey, moderate risk in medium grey, and high risk in dark grey. Upon comparing predicted 30-d mortality risks using Logistic, Harris, and ML-based calculators, we observed some differences across these tools. Critical differences between the extremes (low risk vs high-risk) were rare, occurring in 1 patient (ID# 17).

**Table 5:** Comparison of predicted 30-day mortality risk using classic, Harris, and machine learning (ML) calculators for 20 orthopedic patients.

**ID#**	**Age**	**Sex**	**Mortality**	**Patient descriptor**	**Classic pars (10 vars)**	**Classic full (16 vars)**	**Harris (16 vars)**	**ML (9 vars)**	**ML (15 vars)**	**ML (28 vars)**	
**1**	74	1	0	Diabetic, spinal anesthesia, high BUN-creatinine, mild anemia, ASA class 3, overweight, mild anemia	3	2	2	2	3	3	low risk <4
**2**	70	0	0	Diabetic, general anesthesia, obese, COPD, ASA 4,	5	5	4	4	6	7	intermediate risk 4-8
**3**	76	1	0	General anesthesia, SIRS, anemic, ASA 3, normal BMI	3	3	3	3	3	3	high risk ≥8
**4**	78	0	0	Bleeding disorder, high INR, spinal anesthesia, ASA 3, normal BMI	3	3	3	3	3	3	
**5**	81	1	0	Bleeding disorder, general anesthesia, ASA 3	3	4	3	2	1	1	
**6**	81	0	0	Diabetic, partially dependent, ASA 3, overweight, mild anemia	15	12	6	8	11	10	
**7**	86	1	0	General anesthesia, anemic, ASA 3	4	4	3	5	2	2	
**8**	85	0	0	Partially dependent, general anesthesia, ASA 3	12	11	9	9	13	13	
**9**	90	1	0	General anesthesia, age 90, ASA 3, emergency, mild anemia	7	6	5	8	4	5	
**10**	90	0	0	Spinal anesthesia, age 90+, COPD, ASA 4, overweight	13	12	10	16	11	15	
**11**	73	1	1	General anesthesia, COPD + dyspnea upon moderate exertion, SIRS, ASA 4	4	4	5	6	9	10	
**12**	73	0	1	Spinal, 90+, COPD, ASA 4, overweight, bleeding disorder, SIRS, low platelets, ASA 4, underweight, anemia	7	8	7	7	9	7	
**13**	75	1	1	Partially dependent, diabetes, general anesthesia, dialysis, steroids, SIRS, anemic/high INR, ASA 4	8	9	8	13	21	19	
**14**	79	0	1	dialysis on steroids, general anesthesia, anemic, emergency, ASA 4,	10	11	5	11	11	10	
**15**	80	1	1	General anesthesia, bleeding disorder (low platelets/high INR), SIRS, anemic, ASA 4	4	5	4	14	11	10	
**16**	81	0	1	General anesthesia. COPD, ascites, high creatinine, INR, mild anemia, ASA 4	18	40	6	9	13	15	
**17**	88	1	1	General anesthesia, ASA 4	3	3	3	10	4	5	
**18**	88	0	1	Diabetic, spinal anesthesia, COPD, SIRS, high creatinine, emergency, ASA 3, overweight,	8	9	9	6	9	10	
**19**	90	1	1	General anesthesia, partially dependent, ASA 3,	11	11	9	10	9	8	
**20**	90	0	1	General anesthesia, bleeding disorder (high INR), ASA 3, overweight,	8	10	8	9	15	15	
				Average	7.49	8.57	5.59	7.73	8.43	8.63	
				Average dead	8.15	10.89	6.42	9.54	11.07	11.15	
				Average alive	6.84	6.26	4.76	5.91	5.79	6.11	

Sex is coded as 0 = male and 1 = female; mortality status is coded as 0 = alive and 1 = deceased.

Abbreviations: ASA, American Society of Anesthesiologists Physical Status Classification; COPD, chronic obstructive pulmonary disease, INR, International Normalized Ratio; SIRS, Systemic Inflammatory Response Syndrome.

Among the first 10 surviving patients, the low-risk category (light grey) predominated in 5/10 patients (ID# 1, 3, 4, 5, 7), whereas among the 10 deceased patients, the high-risk category (red) was most common in 6/10 patients (ID# 13, 14, 16, 18, 19, and 20). Importantly, when examining predicted mortality risk categories based on dead and alive, almost all deceased patients consistently fell into the high-risk (red) category across all calculators (with exception of ID# 11, 12, 15, and 17), while survivors predominantly occupied the low (light grey) or intermediate-risk (medium grey) category, with few in the high risk (red, ID# 6, 8, and 10). The latter 3 were older, above age 80, and had a poorer functional health status and high ASA score, strong predictors across all calculators.

### Online calculator

The 15-variable preoperative ML model was selected for deployment based on a careful balance between predictive performance and clinical practicality. While larger models demonstrated slightly higher AUCs, the 15-variable model maintained a high level of accuracy, demonstrating robust performance across multiple validation datasets. This streamlined ML model was chosen specifically for its parsimonious design, minimizing the number of variables required for input while preserving its strong discriminatory power. Reducing the number of variables makes the model more accessible and user-friendly, particularly in fast-paced clinical environments, where time and data collection may be limited. Moreover, the 15-variable model strikes an optimal compromise between complexity and utility, making it highly feasible for real-world implementation without sacrificing critical performance. The model’s simplicity enhances its potential for widespread adoption by clinicians, while still providing reliable risk predictions. Given these advantages, it was deemed the most suitable for deployment as a public-facing web tool, offering clinicians an efficient, accessible, and accurate tool for preoperative risk assessment. This online calculator can be accessed here: https://trad-hipfracture-mortalitypredictor.streamlit.app/. The online calculator uses the top 15 predictors from the preoperative pool shown in the Table 4: age, sex, height, weight, ASA class, CPT code, functional status, preoperative sepsis, history of severe COPD, and preoperative laboratory values (hematocrit, platelet count, WBC count, sodium, BUN, and INR). To note that the inputs of this calculator are still collected in recent ACS-NSQIP releases. The calculator therefore remains operational with present-day NSQIP variables and does not depend on retired fields. In the external validation cohort (*n* = 47 276), the deployed model (on-line calculator) demonstrated good calibration. The calibration intercept was −0.145 (95% CI: −0.258 to −0.033), indicating a slight overall overestimation of risk. The calibration slope was 0.977 (95% CI: 0.932-1.022), suggesting minimal evidence of overfitting. The calibration plot (Figure S4) showed good agreement between predicted and observed risks across deciles.

## Discussion

This validation study underscores the accuracy of the 30-d mortality models for post-hip fracture surgery developed by each of Rhayem et al.^5^ and Trad et al.,^6^ facilitating more informed decision-making based on personalized risk estimates. It also provides a more comprehensive comparison to another 30-d mortality post hip fracture surgery calculator developed using the same database. It thus affords clinicians, the evidence needed to select the model and calculator of choice, with a deployable version for use at point of care, to inform decision making in the relevant population. As NSQIP evolves, we will track variable dictionary changes updating the tool accordingly.

### Key findings

While the baseline characteristics of the 2011-2017 and 2018-2020 cohorts were generally similar, surgical practice evolved during this period. Applying the logistic and the ML models to the later cohort resulted in minor variations in AUCs that fall within the 99% CIs and reflect the inherent variability that comes with applying models to new clinical populations. The overall performance remains consistently robust in comparison to the original training dataset. The preoperative models have broadly similar performance, with only slight variations among parsimonious and full models, suggesting that simpler models achieve nearly equivalent predictive accuracy to more complex ones (Figure 2A). Incorporating on-postoperative variables significantly enhances predictive performance for all models, reinforcing the value of early postoperative data in refining mortality risk predictions (Figure 2B).

Machine learning models generally outperformed conventional logistic regression, consistently showing significantly higher AUC values in almost every direct comparison except for the preoperative parsimonious. The possible explanation is that in the 10 variable (parsimonious) models, both ML and logistic regression were given a limited set of highly informative, predominantly linear predictors. With such reduced complexity, logistic regression was able to capture almost all of the relevant information. This left little opportunity for ML to leverage its usual strength of uncovering complex nonlinear interactions, and consequently, their AUCs were similar.

### Comparative evaluation with Harris et al.

Our models differ from the study by Harris et al.^8^ We specifically focused on individuals aged 65 and older, whereas Harris et al. included a broader age range (≥18 yr, with 92% >70 yr) and, unlike our exclusion of cancer patients, he incorporated active cancer as a predictor. When the preoperative model from Harris et al. was applied to our more recent 2018-2020 cohort, its performance slightly decreased (0.76-0.749) likely because patient demographics and hip-fracture patterns have evolved. Our simpler preoperative models, despite using fewer predictors, showed comparable predictive accuracy in the same contemporary cohort, suggesting better clinical utility and efficiency.

### Comparison of major predictors by order of importance

Age, functional status, and ASA consistently emerged as top five predictors across all pre and Postoperative models, both logistic and ML, underscoring their importance as indicators of frailty and physiological reserve. Sex was among the predictors in almost all full models, and this is further supported by the finding that most variables differed significantly between males and females (Table 1). Classic logistic models prioritized easily interpretable clinical factors. In the full and parsimonious preoperative model, age was followed by functional health status, gender, and pre-op creatinine. This aligns with established surgical understanding: older, sicker patients with kidney issues face higher risks after hip fracture surgery. For postoperative classic logistic models, the most important factors shifted to early postoperative complications. Unplanned intubation was the leading predictor, followed by acute infections (pneumonia) and cardiopulmonary events (cardiac arrest, MI).

In contrast, ML preoperative models ranked anthropometric data (weight and height) higher than lab values, consistent with findings linking both under- and over-nutrition to increased peri-operative mortality. Machine learning algorithms also account for complex, nonlinear relationships, potentially explaining the significance of weight-related factors despite their lower ranking in traditional regressions. For the ML postoperative models, the preoperative variables age, ASA, and functional health status were the top three. Overall, our findings show that classic regressions offer transparency and clinical interpretability, while ML models may capture nuanced body composition effects and nonlinear interactions.

### Case-based comparison of 20 patients

Our exploratory, color-coded comparison of 20 randomly selected patients (10 survivors and 10 non-survivors) underscored the practical concordance of the three calculators. In survivors, light grey cells (<4% risk) dominated; in non-survivors, dark grey cells (>8% risk) were most frequent. One patient showed a low-vs-high mismatch between calculators (ID# 17), indicating that extreme discordance is rare and that, in most cases, any of the three tools points clinicians in the same clinical direction. In this case of postoperative death where the predictive models disagreed, the ML model accurately identified the patient as high-risk, whereas the logistic regression model incorrectly classified it as low risk. A detailed review of NSQIP variables revealed the basis for this divergence. While only anesthesia type and ASA score were key variables, the ASA score’s significantly predictive power within the ML model likely explains its superior precision in predicting mortality. Differences in the mortality risk across ML-9 vs ML-15/ML-28 for patient ID#17 are expected, because the models use different numbers and types of predictors, and that the more feature-rich models may attenuate or amplify risk depending on the added variables. Moreover, the mean predicted risk for the deceased group fell in the high-risk (red) band for most patients across all models, whereas the surviving group averaged in the low (light grey) and intermediate (medium grey) band, with 3 exceptions (ID# 6, 8, and 10). Altogether, these findings suggest that the calculators are clinically coherent when applied at the bedside.

### Comparison to other studies

Several studies have explored the comparative performance of logistic regression and ML models in predicting outcomes after hip fracture. Michelsen et al. compared prospectively ML (boosted decision trees) and logistic regression models for predicting “medical” morbidity (length of stay > 4 d or 90-d readmission) after fast-track total hip and knee arthroplasty in training and testing sets. Using 33 preoperative variables from a large Danish cohort (*n* = 21 926), the ML model demonstrated a slight improvement in predictive performance (eg, AUROC 76.3% vs 74.7%) over logistic regression. Notably, a more parsimonious ML model (using the top 10 variables) outperformed the full logistic regression model, with an AUC of 0.759 vs 0.738.^11^

In a Taiwanese retrospective cohort of 286 elderly patients undergoing hip fracture surgery, an artificial neural network (ANN) model significantly outperformed logistic regression in predicting 1-yr mortality, with higher AUC values in both training and testing datasets (ANN AUC: 0.998 and 0.949 vs LR AUC: 0.938 and 0.784, respectively). The authors suggested that ANNs may better handle complex nonlinear interactions among variables, in which traditional regression methods might miss.^12^ Finally, a recent systematic review sought to compare the predictive accuracy of ML models against traditional statistical methods, specifically multivariable linear or logistic regression, for estimating postoperative mortality in patients with hip fracture. The findings indicate that ML models achieved a mean AUC of 0.84, which was numerically higher than the 0.79 mean AUC observed for logistic regression models. However, this difference did not reach statistical significance (*p* = .09).^13^

### Limitations and advantages

Several limitations warrant consideration. Our validation exercise used the NSQIP dataset, which is susceptible to coding errors, and only tracks 30-d mortality post-surgery. NSQIP-to-NSQIP validation is expected to show broadly similar baseline characteristics and should be interpreted as a registry-based validation rather than definitive external validation; confirmation in independent, non-NSQIP cohorts is needed before broad generalization. Its applicability to other countries, particularly low- and middle-income countries, is unknown. Changes in NSQIP data collection after 2020 did not allow us to extend our validation dataset to include patients beyond 2020. The selected small sample size of patients, to assess model prediction for mortality, vs actual outcomes across all calculators, restricts the generalizability of our case-based analysis. While it was randomly selected and balanced for key demographics, it may have missed less common presentations but could be tested in the future using a larger sample size. Risk stratification for the case-based analysis was based on cut-offs for mortality risk estimates derived from patients evaluated post cardiac surgery, and thus, these categories may not necessarily apply to mortality risk post-hip fracture surgery. We did not have the opportunity to compare the performance of our calculators to the NSQIP online surgical risk calculator because the beta estimates for that model are not published NSQIP Risk Calculator. We note, however, that the NSQIP online mortality risk calculator is not specific for mortality post-hip fracture surgery.

This study has important advantages. The NSQIP database includes 707 hospitals delivering care to adult patients across the United States, thus its relevance to this population. Importantly, it is anchored in real time data obtained from patients’ medical records, rather than being an administrative claims databases or single center studies,^13^ which is the case for the majority of datasets used to assess impact of clinical care on major health outcomes worldwide. It thus allows a unique risk-based analysis in general, and a case-based selection for clinical validation. Indeed, this was demonstrated in our random selection of 20 patients, where we targeted a range of risk profiles using the clinical variables available in the dataset. We’ve built our predictions on one of the largest hip-fracture cohort ever reported, ensuring statistical power and real-world applicability. We provide two calculators: one using preoperative variables available at admission and another using postoperative 30-d course variables (which may include events occurring after discharge), taking into account complications during admission that may impact 30-d mortality. We have established that parsimonious versions only using 10-15 easily gathered clinical variables maintain nearly identical accuracy to full models, simplifying bedside implementation. We also provide a freely available web calculator, a comprehensive solution that previous studies did not offer. Indeed, other published hip-fracture mortality models commonly suffer from critical limitations^13^: they typically rely on small, single-center samples; use administrative claims databases lacking clinical granularity; provide predictions at only one peri-operative time point, require cumbersome, extensive data inputs and lack a web base calculator. These disadvantages significantly reduce their clinical applicability and generalizability.

### Conclusion

The initial models we developed^5^^,^^6^ provide robust 30-d mortality risk estimates, enhancing the precision of informed consent and shared decision-making. However, even elevated risk projections should not serve as grounds for surgical exclusion. Many patients classified as high risk exhibit extended survival beyond model predictions, underscoring the necessity for individualized treatment discussions, and the clinical uncertainties remain.

Traditional and modern ML risk calculators offer distinct yet valuable benefits for surgical decisions. While classic tools provide transparency and simplicity, ML models enhance risk prediction by analyzing more data and complex relationships. Integrating both into clinical practice via electronic medical records systems or applications creates a strong foundation for identifying high-risk patients, guiding optimization, and supporting shared decision-making. Future research should focus on prospective validation of these tools and to show how they may impact clinical practice and patient outcomes across various populations. Continuous model updates will be crucial to account for changes in coding, techniques, and patient populations. Combining the clarity of classic scores with the adaptability of ML promises more precise, actionable, and equitable peri-operative risk assessment.

## Supplementary Material

Appendix_March_22_ziag079

## Data Availability

Data were obtained from the American College of Surgeons NSQIP datasets (2018-2020). Restrictions apply to their availability; they are not publicly available but may be requested from the ACS NSQIP program.

## References

[1] GBD 2019 Fracture Collaborators . Global, regional, and national burden of bone fractures in 204 countries and territories, 1990-2019: a systematic analysis from the Global Burden of Disease Study 2019. Lancet Healthy Longev. 2021;2(9):e580-e592. 34723233 10.1016/S2666-7568(21)00172-0 PMC8547262

[2] Tian C, Shi L, Wang J, et al. Global, regional, and national burdens of hip fractures in elderly individuals from 1990 to 2021 and predictions up to 2050: a systematic analysis of the Global Burden of Disease Study 2021. Arch Gerontol Geriatr. 2025;133:105832. 10.1016/j.archger.2025.105832 40112671

[3] Veronese N, Maggi S. Epidemiology and social costs of hip fracture. Injury. 2018;49(8):1458-1460. 10.1016/j.injury.2018.04.015 29699731

[4] International Osteoporosis Foundation . Osteoporosis Facts and Statistics. Accessed June 20, 2025. https://www.osteoporosis.foundation/facts-statistics.

[5] Rhayem C, Ghosn A, Issa ZA, et al. Secular trends in hip fracture mortality and predictors of mortality from the NSQIP database. J Clin Endocrinol Metab. 2025;110(11):3210-3219. 10.1210/clinem/dgaf087 39980170 PMC12527425

[6] Trad F, Isber B, Yammine R, et al. Parsimonious and explainable machine learning for predicting mortality in patients post hip fracture surgery. Sci Rep. 2025;15(1):22922. 10.1038/s41598-025-98713-6 40596420 PMC12216216

[7] Ramspek CL, Jager KJ, Dekker FW, et al. External validation of prognostic models: what, why, how, when and where? Clin Kidney J. 2020;14(1):49-58. 33564405 10.1093/ckj/sfaa188 PMC7857818

[8] Harris AHS, Trickey AW, Eddington HS, et al. A tool to estimate risk of 30-day mortality and complications after hip fracture surgery: accurate enough for some but not all purposes? A study from the ACS-NSQIP database. Clin Orthop Relat Res. 2022;480(12):2335-2346. 10.1097/CORR.0000000000002294 35901441 PMC10538935

[9] DeLong ER, DeLong DM, Clarke-Pearson DL. Comparing the areas under two or more correlated receiver operating characteristic curves: a nonparametric approach. Biometrics. 1988;44(3):837-845. 10.2307/2531595 3203132

[10] Al-Azizi K, Shih E, DiMaio JM, et al. Assessment of TVT and STS risk score performances in patients undergoing transcatheter aortic valve replacement. J Soc Cardiovasc Angiogr Interv. 2023;2(3):100600. 10.1016/j.jscai.2023.100600 39130722 PMC11308024

[11] Michelsen C, Jørgensen CC, Heltberg M, et al. Machine-learning vs. logistic regression for preoperative prediction of medical morbidity after fast-track hip and knee arthroplasty-a comparative study. BMC Anesthesiol. 2023;23(1):391. 10.1186/s12871-023-02354-z PMC10685559 38030979

[12] Lin CC, Ou YK, Chen SH, Liu YC, Lin J. Comparison of artificial neural network and logistic regression models for predicting mortality in elderly patients with hip fracture. Injury. 2010;41(8):869-873. 10.1016/j.injury.2010.04.023 20494353

[13] Lex JR, Di Michele J, Koucheki R, et al. Artificial intelligence for hip fracture detection and outcome prediction: a systematic review and meta-analysis. JAMA Netw Open. 2023;6(3):e233391. 10.1001/jamanetworkopen.2023.3391 36930153 PMC10024206

