# Supplementary material for: Are machine learning models superior to logistic regression models to predict 30-day mortality post-hip fracture surgery?
Source: JBMR Plus. 2026 Apr 28;10(7):ziag079. doi: 10.1093/jbmrpl/ziag079 (PMC13267897; doi:10.1093/jbmrpl/ziag079)
Supplement: Appendix_March_22_ziag079 [file appendix_march_22_ziag079.docx]

**Appendix**

We selected the below specific 5 current procedural terminology (CPT) codes for hip surgeries:

1. CPT 27125: partial hip hemiarthroplasty

2. CPT 27236: open reduction and internal fixation (ORIF) of a femoral neck fracture

3. CPT 27244: ORIF of an intertrochanteric, peritrochanteric, or subtrochanteric femoral fracture

4. CTP 27245: intramedullary fixation of an intertrochanteric, peritrochanteric, or subtrochanteric femoral fracture

5. CPT 27130: total hip arthroplasty

After selecting the appropriate CPT codes for hip surgeries, we selected the appropriate international classification of diseases (ICD) codes for hip fractures at the following sites: femoral neck, intertrochanteric, subtrochanteric and peritrochanteric. For those who have ICD 10, we selected the following cases: age related osteoporosis (M80.051A, M80.051G, M80.051K, M80.051P, M80.052A, M80.052G, M80.052K, M80.052P, M80.059A, M80.059G, M80.059K, M80.059P), S72.0x for femoral neck, S72.1x for intertrochanteric, and S72.2x for subtrochanteric fractures. For those who have ICD 9, we selected the following cases: ICD 820 and 820.xx. We excluded atypical and pathologic fractures using ICD-10 M84.75 (Atypical femoral fracture) and M84.6 (Pathological fracture in other disease).

We excluded patients with preoperative disseminated cancer using the NSQIP preop variables (disseminated cancer, recent chemo and radiotherapy).

**Figure S1.** DeLong Test P-values between different models


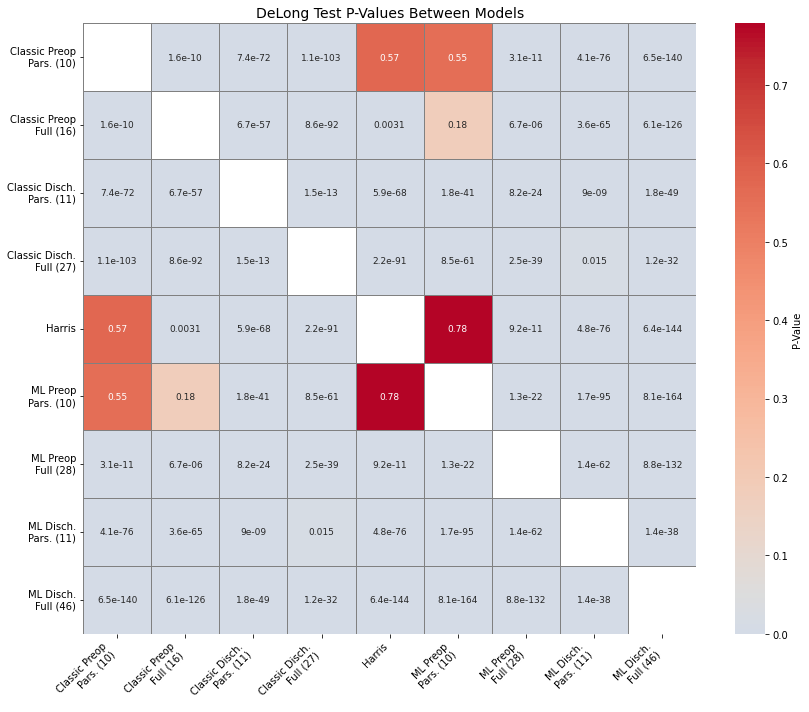


**Figure S2. Patient Flow diagram**

**Table S1.**

| **Category** | **Variables (names only)** | **Timing** |
| --- | --- | --- |
| **Preoperative variables** | Age of patient (years) |  |
|  | Pre-operative hematocrit (%) | Days from preop HCT to OR  Mean (SD) 0.49 (2.3) |
|  | Pre-operative serum creatinine (mg/dL) | Days from preop Creat to OR  Mean (SD) 0.52 (2.6) |
|  | Race (Black/African American; White; Others; Unknown) |  |
|  | BMI category (<18.5; 18.5–24.9; 25–29.9; ≥30 kg/m²) |  |
|  | Smoker within 1 year (Yes/No) |  |
|  | Diabetes (Yes/No) |  |
|  | Hypertension requiring medication (Yes/No) |  |
|  | History of severe COPD (Yes/No) |  |
|  | Congestive heart failure within 30 days before surgery (Yes/No) |  |
|  | Bleeding disorders (Yes/No) |  |
|  | Functional health status prior to surgery (Independent / Partially dependent / Totally dependent) |  |
| **Intraoperative variables** | Total operation time (minutes) |  |
|  | Anesthesia type (General / Neuraxial) |  |
|  | Type of surgery (IM fixation; Partial hip arthroplasty; ORIF femoral neck fracture; ORIF inter/sub/peri-trochanteric femoral fracture; Total hip arthroplasty) |  |
| **Postoperative variables** | Occurrence of unplanned intubation | Not available |
|  | Postoperative CVA/stroke | Not available |
|  | Postoperative myocardial infarction | Not available |
|  | Postoperative pneumonia | Not available |

**Table S2: AUC and CI for the Different Models for Using NSQIP 2011–2017 and NSQIP 2018–2020 datasets.**

| **Model (N variables)** | **NSQIP 2011–2017**  **N subjects 73,291** | **NSQIP 2018–2020**  **N subjects 47,276** |
| --- | --- | --- |
| **Classic Preoperative Parsimonious** (10) | 0.739 (0.732, 0.746) | 0.748 (0.736, 0.760) |
| **Classic Preoperative Full** (16) | 0.742 (0.735, 0.748) | 0.756 (0.743, 0.768) |
| **Classic Postoperative Parsimonious** (11) | 0.800 (0.793, 0.806) | 0.817 (0.806, 0.828) |
| **Classic Postoperative Full** (27) | 0.813 (0.807, 0.819) | 0.829 (0.819, 0.839) |
| **ML Preoperative** **Parsimonious** (10) | 0.771 (0.729, 0.817) | 0.751 (0.739, 0.766) |
| **ML Preoperative Full** (28) | 0.783 (0.743, 0.823) | 0.772 (0.760, 0.785) |
| **ML Postoperative Parsimonious** (11) | 0.860 (0.826, 0.889) | 0.838 (0.828, 0.849) |
| **ML Postoperative Full** (46) | 0.885 (0.853, 0.916) | 0.865 (0.856, 0.875) |
| **Harris* et al** (15) | 0.760 (0.750, 0.760) | 0.749 (0.737, 0.761) |

**Table S3. Missing data per predictor**

| **Variable** | **Missing (n)** | **Missing (%)** |
| --- | --- | --- |
| Age of patient (years) | 0 | 0.00 |
| Preoperative hematocrit (%) | 400 | 0.58 |
| Preoperative serum creatinine (mg/dL) | 0 | 0.00 |
| Total operation time (minutes) | 0 | 0.00 |
| Race | 0 | 0.00 |
| BMI category (BMI_c) | 9,029 | 13.18 |
| Smoker within 1 year | 0 | 0.00 |
| Diabetes | 0 | 0.00 |
| Hypertension requiring medication | 0 | 0.00 |
| History of severe COPD | 0 | 0.00 |
| Congestive heart failure in 30 days before surgery | 0 | 0.00 |
| Bleeding disorders | 0 | 0.00 |
| Functional health status prior to surgery | 0 | 0.00 |
| Anesthesia type (Anesth_Calc) | 25 | 0.04 |
| Type of surgery | 0 | 0.00 |
| Pre-op white blood cell count (10³/µL) | 422 | 0.62 |
| ASA classification | 132 | 0.19 |
| Pre-op INR | 9,931 | 14.49 |

**Equation of the Harris et al. (2022) Published Comparator (LASSO Logistic Regression Coefficients)**

EXP(-3.53 + (-0.5*Female) + (0.34*age_h_dummy_4) + (0.57*age_h_dummy_5) + (1.09*age_h_dummy_6) + (0.31*bmi_h2_1) + (-0.14*bmi_h2_3) + (-0.17*bmi_h2_4) + (-0.01*bmi_h2_5) + (0.66*wtloss_calc) + (0.05*Diabetes_Dummy_1) + (-0.1*smoke_c) + (0.67*fnstatus_Dummy_14) + (1.03*fnstatus_Dummy_15) + (0.22*Dyspnea_Dummy_2) + (0.53*Dyspnea_Dummy_1) + (0.4*HXCOPD_calc) + (0.63*HXCHF_Calc) + (0.39*PRsep_Dummy_2) + (0.54*PRsep_Dummy_3) + (1.39*PRsep_Dummy_4) + (0.03*Steroid_Calc) + (0.2*Bleeddis_Calc) + (0.56*Dialysis_c) + (-0.05*CPTnew_27130))

STROBE Statement—checklist of items that should be included in reports of observational studies

|  | **Item No.** | **Recommendation** | **Page  No.** | **Relevant text from manuscript** |
| --- | --- | --- | --- | --- |
| **Title and abstract** | 1 | (*a*) Indicate the study’s design with a commonly used term in the title or the abstract | 2 | NSQIP dataset, a cohort |
|  |  | (*b*) Provide in the abstract an informative and balanced summary of what was done and what was found | 2-3 |  |
| **Introduction** | | | |  |
| Background/rationale | 2 | Explain the scientific background and rationale for the investigation being reported | 5 | The background outlines the burden of hip fractures and the need for better prediction tools. |
| Objectives | 3 | State specific objectives, including any prespecified hypotheses | 6 | All aims are listed after the introduction |
| **Methods** | | | |  |
| Study design | 4 | Present key elements of study design early in the paper | 7-10 | All the key elements are listed in the methods |
| Setting | 5 | Describe the setting, locations, and relevant dates, including periods of recruitment, exposure, follow-up, and data collection | 7-10 | Listed in the methods section |
| Participants | 6 | (*a*) *Cohort study*—Give the eligibility criteria, and the sources and methods of selection of participants. Describe methods of follow-up  *Case-control study*—Give the eligibility criteria, and the sources and methods of case ascertainment and control selection. Give the rationale for the choice of cases and controls  *Cross-sectional study*—Give the eligibility criteria, and the sources and methods of selection of participants | 7 | the eligibility criteria, and the sources and methods of selection of participants are listed in page 7 |
|  |  | (*b*) *Cohort study*—For matched studies, give matching criteria and number of exposed and unexposed  *Case-control study*—For matched studies, give matching criteria and the number of controls per case |  |  |
| Variables | 7 | Clearly define all outcomes, exposures, predictors, potential confounders, and effect modifiers. Give diagnostic criteria, if applicable | 7-10 | all outcomes, exposures, predictors, potential confounders, and effect modifiers listed in the methods section |
| Data sources/ measurement | 8* | For each variable of interest, give sources of data and details of methods of assessment (measurement). Describe comparability of assessment methods if there is more than one group | *7-10* | NSQIP database |
| Bias | 9 | Describe any efforts to address potential sources of bias | 7-10 | NSQIP database |
| Study size | 10 | Explain how the study size was arrived at | 7-10 | NSQIP database |

Continued on next page

| Quantitative variables | 11 | Explain how quantitative variables were handled in the analyses. If applicable, describe which groupings were chosen and why | 7-10 | Listed in the methods section |
| --- | --- | --- | --- | --- |
| Statistical methods | 12 | (*a*) Describe all statistical methods, including those used to control for confounding | 8-10 | For each outcome, the statistical analysis was listed |
|  |  | (*b*) Describe any methods used to examine subgroups and interactions | Not applicable |  |
|  |  | (*c*) Explain how missing data were addressed |  |  |
|  |  | (*d*) *Cohort study*—If applicable, explain how loss to follow-up was addressed  *Case-control study*—If applicable, explain how matching of cases and controls was addressed  *Cross-sectional study*—If applicable, describe analytical methods taking account of sampling strategy | 7 | No missing data |
|  |  | (*e*) Describe any sensitivity analyses |  | Not applicable |
| Results | | | | |
| Participants | 13* | (a) Report numbers of individuals at each stage of study—eg numbers potentially eligible, examined for eligibility, confirmed eligible, included in the study, completing follow-up, and analysed | 10 | Numbers of individuals at each stage of study, examined for eligibility, confirmed eligible, included in the study, completing follow-up, and analysed |
|  |  | (b) Give reasons for non-participation at each stage |  | Not applicable |
|  |  | (c) Consider use of a flow diagram |  | Not applicable |
| Descriptive data | 14* | (a) Give characteristics of study participants (eg demographic, clinical, social) and information on exposures and potential confounders | 10 | Results section and Table 1 describe characteristics |
|  |  | (b) Indicate number of participants with missing data for each variable of interest | 7 | No missing data |
|  |  | (c) *Cohort study*—Summarise follow-up time (eg, average and total amount) | 6 | 30 day mortality |
| Outcome data | 15* | *Cohort study*—Report numbers of outcome events or summary measures over time | *11-16* | numbers of outcome events or summary measures over time listed |
|  |  | *Case-control study—*Report numbers in each exposure category, or summary measures of exposure |  |  |
|  |  | *Cross-sectional study—*Report numbers of outcome events or summary measures |  |  |
| Main results | 16 | (*a*) Give unadjusted estimates and, if applicable, confounder-adjusted estimates and their precision (eg, 95% confidence interval). Make clear which confounders were adjusted for and why they were included | 11-16 | Table 2- table 3 |
|  |  | (*b*) Report category boundaries when continuous variables were categorized |  | Not applicable |
|  |  | (*c*) If relevant, consider translating estimates of relative risk into absolute risk for a meaningful time period |  | Not applicable |

Continued on next page

| Other analyses | 17 | Report other analyses done—eg analyses of subgroups and interactions, and sensitivity analyses |  |  |
| --- | --- | --- | --- | --- |
| Discussion | | | | |
| Key results | 18 | Summarise key results with reference to study objectives |  |  |
| Limitations | 19 | Discuss limitations of the study, taking into account sources of potential bias or imprecision. Discuss both direction and magnitude of any potential bias |  |  |
| Interpretation | 20 | Give a cautious overall interpretation of results considering objectives, limitations, multiplicity of analyses, results from similar studies, and other relevant evidence |  |  |
| Generalisability | 21 | Discuss the generalisability (external validity) of the study results |  |  |
| Other information | |  | | |
| Funding | 22 | Give the source of funding and the role of the funders for the present study and, if applicable, for the original study on which the present article is based |  |  |

*Give information separately for cases and controls in case-control studies and, if applicable, for exposed and unexposed groups in cohort and cross-sectional studies.

**Note:** An Explanation and Elaboration article discusses each checklist item and gives methodological background and published examples of transparent reporting. The STROBE checklist is best used in conjunction with this article (freely available on the Web sites of PLoS Medicine at http://www.plosmedicine.org/, Annals of Internal Medicine at http://www.annals.org/, and Epidemiology at http://www.epidem.com/). Information on the STROBE Initiative is available at www.strobe-statement.org.

**Figure S3. Calibration plot – Logistic Preoperative parsimonious model (external validation).**


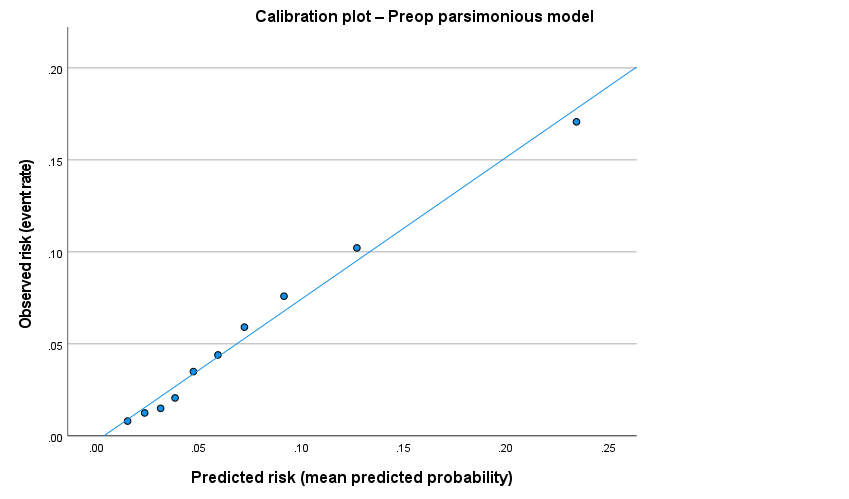


Observed event rates (mean of the binary outcome) are plotted against mean predicted probabilities within deciles of predicted risk in the external validation cohort (n = 47,276).

**Figure S4. Calibration plot – Deployed ML model (external validation).**


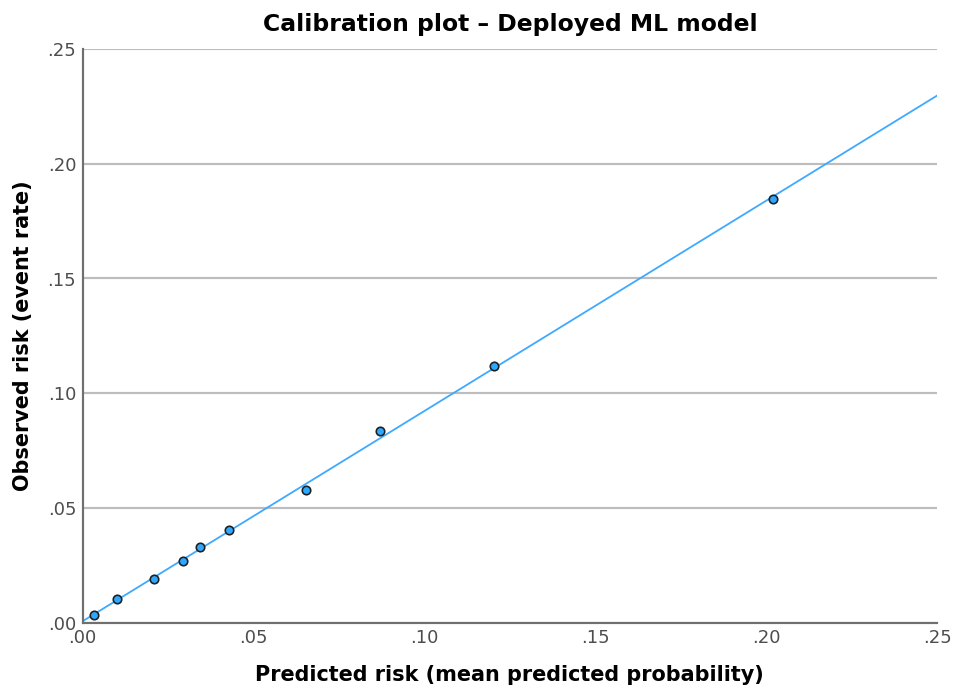


Observed event rates (mean of the binary outcome) are plotted against mean predicted probabilities within deciles of predicted risk in the external validation cohort (n = 47,276).
